# Supplementary material for: A Global Screen for Assembly State Changes of the Mitotic Proteome by SEC-SWATH-MS
Source: Cell Syst. 2020 Feb 26;10(2):133–155.e6. doi: 10.1016/j.cels.2020.01.001 (PMC7042714; doi:10.1016/j.cels.2020.01.001)

Q9H8M7 | F188A_HUMAN | FAM188A C10orf97 CARP DERP5 MSTP126 My042
Monomer MW [kDa]: 49.725 Monomer expected elution fraction: 46

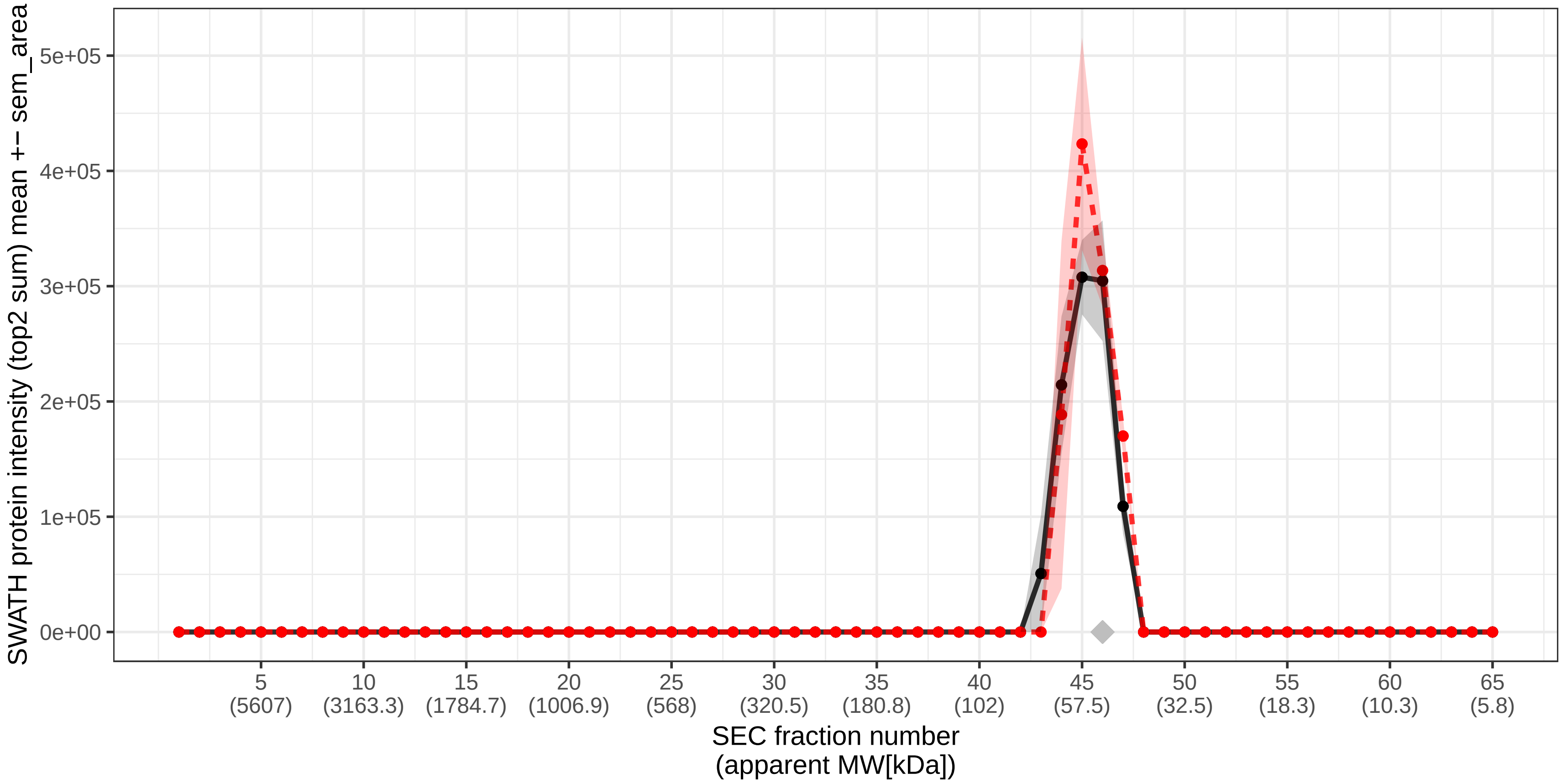

Supplement: Data S1. SEC-SWATH-MS Protein Chromatograms, Related to Figure 1 [file mmc6.zip › SECchrom_Q9H8M7_F188A_HUMAN_FAM188A_C10orf97_CARP_DERP5_MST.pdf]
